# Risk of Parkinson Disease Among Patients With Restless Leg Syndrome

**DOI:** 10.1001/jamanetworkopen.2025.35759

**Published:** 2025-10-06

**Authors:** Myeonghwan Bang, Dougho Park, Jong Hun Kim, Hyoung Seop Kim

**Affiliations:** 1 Department of Physical Medicine and Rehabilitation, National Health Insurance Service Ilsan Hospital, Goyang, Republic of Korea; 2 Medical Research Institute, Pohang Stroke and Spine Hospital, Pohang, Republic of Korea; 3 Department of Neurology, Korea University Ansan Hospital, Ansan, Republic of Korea

## Abstract

**Importance:**

The association between restless leg syndrome (RLS) and Parkinson disease (PD) remains unclear. Clarifying this association and the role of the dopaminergic pathway may improve understanding of the pathophysiology between these 2 diseases.

**Objectives:**

To assess whether RLS is a risk factor for developing PD and whether the dopamine pathway is meaningfully associated with RLS and PD.

**Design, Setting, and Participants:**

This retrospective cohort study used data from the Korean National Health Insurance Service Sample Cohort from 2002 to 2019. Statistical analyses were performed between September 2024 and March 2025. Patients with RLS and PD were identified based on codes from the *International Statistical Classification of Diseases and Related Health Problems, Tenth Revision* and matched to individuals without RLS. For the secondary analysis, the dopamine agonist (DA)–treated group was operationally defined as patients with RLS who received DA during 2 or more distinct clinical visits, while those who did not meet this criterion were classified as the DA-nontreated group.

**Exposures:**

Diagnosis of RLS and treatment with DAs.

**Main Outcomes and Measures:**

Development of PD. To compare the time to PD diagnosis across groups, a restricted mean survival time (RMST) analysis was conducted.

**Results:**

A total of 9919 patients with RLS and 9919 matched controls were included. The mean (SD) age at enrollment was 50.1 (16.3) years in the control group (6225 women [62.8%]) and 50.3 (16.0) years in the RLS group (6225 women [62.8%]). The incidence of PD was 1.0% (99 of 9919) in the control group and 1.6% (158 of 9919) in the RLS group. At the prespecified time horizon of 15 years (τ = 15), the RMST to PD diagnosis was 14.93 years in the control group and 14.88 years in the RLS group, resulting in a difference of −0.05 years (95% CI, –0.07 to –0.03 years). Compared with the control group, the DA-nontreated RLS group (n = 6842) showed a significantly shorter RMST to PD diagnosis (difference, –0.09 years [95% CI, –0.12 to –0.06 years]) and a higher incidence rate (143 of 6842 [2.1%]). The DA-treated RLS group (n = 3077) showed a significantly longer RMST to PD diagnosis (difference, 0.03 years [95% CI, 0.01-0.06 years]) and a lower incidence rate (15 of 3077 [0.5%]).

**Conclusions and Relevance:**

In this cohort study, RLS was associated with an increased risk of developing PD. Furthermore, patients with RLS who were not treated with DAs tended to be at increased risk of developing PD, whereas those who were treated with DAs tended to be at decreased risk compared with the control group. These findings suggest that the pathophysiological connection between RLS and PD may involve mechanisms beyond the dopaminergic pathway.

## Key Points

**Question:**

Is restless leg syndrome (RLS) associated with an increased risk of developing Parkinson disease (PD)?

**Findings:**

In this nationwide population-based cohort study, 9919 patients with RLS showed a statistically significantly higher risk of developing PD than those without RLS. Patients with RLS who were not treated with dopamine agonists showed a shorter time to PD diagnosis, whereas those who were treated with dopamine agonist showed longer time to PD diagnosis.

**Meaning:**

This study suggests that RLS is associated with an increased risk of PD, and the association may involve mechanisms beyond the dopaminergic pathway.

## Teaser

This cohort study uses data from the Korean National Health Insurance Service Sample Cohort to assess whether restless leg syndrome is a risk factor for developing Parkinson disease and whether the dopamine pathway is meaningfully associated with restless leg syndrome and Parkinson disease.

## Introduction

Restless leg syndrome (RLS) is a prevalent disorder characterized by an urge to move the lower limbs, often accompanied by unpleasant and uncomfortable sensations.^1,2,3^ The symptoms tend to be aggravated during periods of rest, which are alleviated during movement. Although RLS may be idiopathic, it can also be associated with other medical comorbidities, such as iron deficiency anemia (IDA), chronic kidney disease, polyneuropathy, diabetes, multiple sclerosis, and cardiovascular diseases.^3,4,5^ Several drugs are used for the treatment of RLS; however, dopamine agonists (DAs) are particularly effective in alleviating symptoms and are thus regarded as the first-line treatment. The pathophysiology of RLS is not yet clearly understood^1,3^; however, it is hypothesized that dopaminergic mechanisms play an important role.^5^

Parkinson disease (PD) is a representative neurodegenerative disorder with an increasing global incidence and a gradually increasing associated social burden. Consequently, identifying risk factors for PD and implementing early interventions are becoming increasingly important. Research on the risk factors and related diseases associated with the development of PD is substantial. Both RLS and PD are treated with dopaminergic agents; numerous studies have been conducted to clarify the association between the 2 disorders.^1,6,7,8^ However, whether RLS is a prodromal symptom of PD or a secondary condition of PD and how the dopaminergic pathway may be the primary connection between the 2 conditions remain unclear.^1,6,8,9,10^ Studies on the development of PD among patients with RLS have been reported; however, because previous studies have focused primarily on male-dominant cohorts, it is difficult to generalize their findings to the general population.^6,9^ This nationwide retrospective cohort study examined whether RLS is associated with an increased risk of PD and explored the potential role of the dopaminergic pathways in this association by stratifying patients with RLS based on DA treatment.

## Methods

### Study Design and Participants

This retrospective cohort was conducted using data from the Korean National Health Insurance Service Sample Cohort (2002-2019). This comprehensive database contains deidentified records for 1 million individuals, representing a 2% stratified random sample of the Korean population. The dataset includes 3 primary components: qualification data (demographic information including age, sex, region, insurance type, and socioeconomic variables such as income level and disability status), regular medical checkup records, and treatment tables containing detailed health care use data.^11^ This study was approved by the institutional review board of the National Health Insurance Service Ilsan Hospital. Given the retrospective nature of the study, the requirement for informed consent was waived. This study was also performed in compliance with the Declaration of Helsinki.^12^ This study followed the Strengthening the Reporting of Observational Studies in Epidemiology (STROBE) reporting guideline for cohort studies.

**Figure 1.**
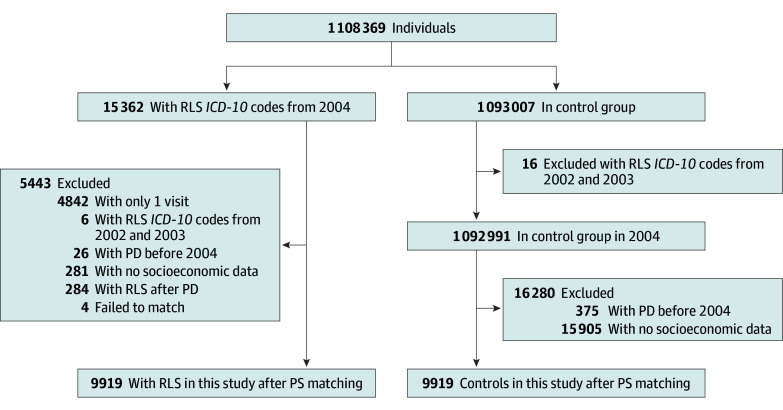
Flowchart of Study Population Selection *ICD-10* indicates the *International Statistical Classification of Diseases and Related Health Problems, Tenth Revision*; PD, Parkinson disease; PS, propensity score; and RLS, restless leg syndrome.

Cases of RLS were identified using the *International Statistical Classification of Diseases and Related Health Problems, Tenth Revision* (*ICD-10*) code G25.8, with diagnostic validity ensured by requiring 2 or more documented RLS diagnoses in the outpatient department (excluding 4842 single-visit cases). To establish an incident RLS cohort, 6 patients diagnosed during 2002 to 2003 were excluded from the study. The exclusion criteria also included the following: preexisting PD diagnosed before 2004 (n = 26), PD diagnosed prior to RLS onset (n = 284), cases lacking socioeconomic data (n = 281), and unmatched individuals (n = 4). The final cohort included 9919 patients with RLS and an equal number of controls matched 1:1 by age, sex, income, region, Charlson Comorbidity Index (CCI) score, and index date (Figure 1).

### Variables and Outcome Definitions

Parkinson disease was defined using *ICD-10* code G20 or code V124 in the claims databases. V-codes are established by the Registration Program for Rare or Intractable Diseases of Korea and are used for copayment claims associated with rare and intractable conditions.^13^

We first compared the risk of developing PD between patients with RLS and the control group. In the second analysis, we classified patients with RLS according to DA treatment and compared the risk of developing PD between the groups. We operationally defined the DA-treated group (RLS group 1) as patients with RLS who had received DAs, such as pramipexole or ropinirole, during at least 2 separate outpatient visits or hospitalizations recorded in the claim database. These patients were considered to have primary RLS that is responsive to DA. In contrast, the DA-nontreated group (RLS group 2) did not meet these criteria and was presumed to consist of patients with secondary RLS cases in which DA treatment was not considered.

This subgroup classification was based on the hypothesis that patients with RLS without identifiable underlying conditions are more likely to have dysfunction of the dopaminergic pathway and, therefore, respond favorably and persistently to DA therapy. In contrast, for patients with comorbidities such as IDA, chronic kidney disease, or polyneuropathy, symptom improvement is more likely to result from correction of the underlying condition, and they may experience limited or no sustained benefit associated with DA therapy.

The CCI, which is considered to adequately estimate 10-year mortality, was used to adjust for comorbidities. In addition to the CCI score, we included a history of sleep disorders (including insomnia, hypersomnia, sleep-related breathing disorders, narcolepsy, sleepwalking, sleep tremors, and nightmares) and IDA as covariates. Comorbidities were defined based on the presence or absence of corresponding disease codes claimed by the Health Insurance Review and Assessment Service.

### Statistical Analysis

Statistical analysis was performed between September 2024 and March 2025. Baseline characteristics were compared using χ^2^ tests for categorical variables and Mann-Whitney *U* tests for continuous variables. Baseline covariate balance was assessed using standardized mean differences, variance ratios, and empirical cumulative distribution function statistics, estimated with the MatchIt package in R, version 4.1.2 (R Project for Statistical Computing).^14^ Standardized mean differences with 95% CIs were calculated using the Hedges *g* for continuous variables, the Cohen method for binary variables, and a Mahalanobis distance-based approach for multilevel categorical variables.^15,16,17^ Cumulative PD incidence was analyzed by Kaplan-Meier survival curves with log-rank tests. Restless leg syndrome was treated as a time-varying exposure, classifying person-time before RLS diagnosis as control and after as exposed. To address baseline confounding, inverse probability of treatment weighting using propensity scores (adjusted for age, sex, income, region, CCI score, body mass index, smoking, alcohol use, sleep disorder, and IDA) with stabilized weights were applied to improve statistical stability. Because the proportional hazards assumption was violated (Schoenfeld residual test; *P* < .001), we estimated the restricted mean survival time (RMST) as a robust alternative to the hazard ratio (eMethods in Supplement 1). Missing data were addressed through complete-case analysis. A 2-tailed *P* < .05 was considered statistically significant. All statistical analyses were conducted using SAS, version 9.4 (SAS Institute Inc).

## Results

### Baseline Characteristics of the Study Population

The study enrolled 9919 individuals in the control group (mean [SD] age, 50.1 [16.3] years; 6225 women [62.8%] and 3694 men [37.2%]) and 9919 individuals in the RLS group (mean [SD] age, 50.3 [16.0] years; 6225 women [62.8%] and 3694 men [37.2%]) (Table 1). There were no significant differences between the 2 groups in terms of age, sex, income level, region of residence, CCI score, and prevalence of IDA. However, statistically significant differences were observed between the RLS group and the control group in alcohol drinking (2894 of 8853 [32.7%] vs 3054 of 8427 [36.2%]; *P* < .001) and the prevalence of sleep disorders (598 [6.0%] vs 388 [3.9%]; *P* < .001) (Table 1; eTable 1 and eFigure in Supplement 1).

**Table 1.** Characteristics of the Study Population

Characteristic	Patients, No./total No. (%)			*P* value^a^
	Overall (N = 19 838)	Control group (n = 9919)	RLS group (n = 9919)	
Age at enrollment, mean (SD), y	50.2 (16.2)	50.1 (16.3)	50.3 (16.0)	.41
Sex				
Male	7388/19 838 (37.2)	3694/9919 (37.2)	3694/9919 (37.2)	>.99
Female	12 450/19 838 (62.8)	6225/9919 (62.8)	6225/9919 (62.8)	
Income level by insurance fee				
0-30th Percentile	4780/19 838 (24.1)	2390/9919 (24.1)	2390/9919 (24.1)	>.99
30th-70th Percentile	7418/19 838 (37.4)	3709/9919 (37.4)	3709/9919 (37.4)	
70th-100th Percentile	7640/19 838 (38.5)	3820/9919 (38.5)	3820/9919 (38.5)	
Region of residence				
Rural	11 042/19 838 (55.7)	5521/9919 (55.7)	5521/9919 (55.7)	>.99
Urban	8796/19 838 (44.3)	4398/9919 (44.3)	4398/9919 (44.3)	
CCI score, mean (SD)	1.30 (1.35)	1.30 (1.35)	1.30 (1.35)	>.99
BMI^b^				
<18.5	586/17 304 (3.4)	293/8439 (3.5)	293/8865 (3.3)	.01^c^
18.5 to <23	6248/17 304 (36.1)	3144/8439 (37.3)	3104/8865 (35.0)	
23 to <25	4226/17 304 (24.4)	2036/8439 (24.1)	2190/8865 (24.7)	
≥25	6244/17 304 (36.1)	2966/8439 (35.1)	3278/8865 (37.0)	
Smoking^b^				
No smoking	12 789/17 282 (74.0)	6259/8428 (74.3)	6530/8854 (73.8)	.33
Quit	1440/17 282 (8.3)	715/8428 (8.5)	725/8854 (8.2)	
Smoking	3053/17 282 (17.7)	1454/8428 (17.2)	1599/8854 (18.0)	
Alcohol^b^				
No drinking	11 332/17 280 (65.6)	5373/8427 (63.8)	5959/8853 (67.3)	<.001^c^
Drinking (≥1 times/wk)	5948/17 280 (34.4)	3054/8427 (36.2)	2894/8853 (32.7)	
Comorbidities				
Sleep disorders^d^	986/19 838 (5.0)	388/9919 (3.9)	598/9919 (6.0)	<.001^c^
Iron deficiency anemia	762/19 838 (3.8)	353/9919 (3.6)	409/9919 (4.1)	.06

Abbreviations: BMI, body mass index (calculated as weight in kilograms divided by height in meters squared); CCI, Charlson Comorbidity Index; RLS, restless leg syndrome.

^a^ Continuous variables by Mann-Whitney *U* test and categorical variables by the χ^2^ test.

^b^ BMI, smoking, and alcohol had missing data; therefore, the total numbers differed.

^c^ Statistically significant.

^d^ Insomnia, hypersomnia, sleep-related breathing disorder, narcolepsy, sleepwalking, sleep tremor, and nightmare.

### Risk of PD Among Patients With RLS

The incidence of PD was 1.0% in the control group (99 of 9919; incidence rate, 6.3/10 000 patient-years) and 1.6% in the RLS group (158 of 9919; incidence rate, 10.1/10 000 patient-years). At the prespecified time horizon of 15 years (τ = 15), the RMST to PD diagnosis was 14.93 years in the control group and 14.88 years in the RLS group, yielding a difference of −0.05 years (95% CI, –0.07 to –0.03 years; *P* < .001).

### Risk of PD Among Patients With RLS According to Use of DAs

**Figure 2.**
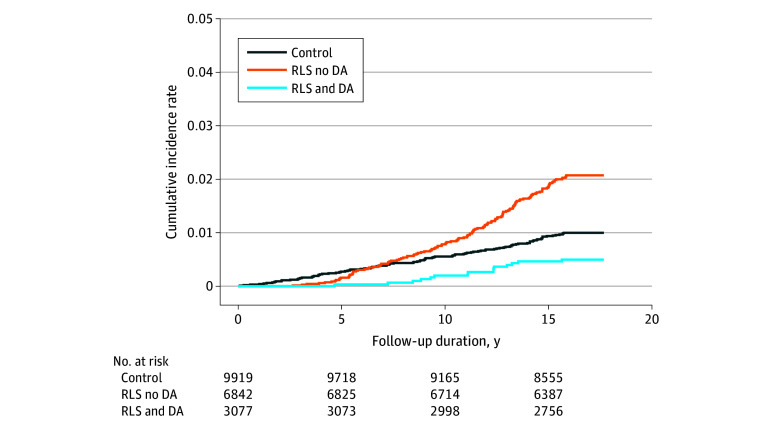
Cumulative Incidence of the Risk of Parkinson Disease DA indicates dopamine agonist; RLS, restless leg syndrome.

Among the patients with RLS, 3077 were allocated to RLS group 1 (DA treated), while 6842 patients were allocated to RLS group 2 (DA nontreated). The characteristics of group 1 and group 2 are summarized in Table 2 and eTable 2 in Supplement 1. During the follow-up period, 15 patients in RLS group 1 developed PD (0.5%; incidence rate, 1.3/10 000 patient-years) compared with 143 patients in RLS group 2 (2.1%; incidence rate, 27.3/10 000 patient-years). The 15-year cumulative incidence of PD was lower in RLS group 1 compared with the control group, whereas it was higher in RLS group 2 than in the control group (Figure 2). Compared with the control group, RLS group 2 had a significantly shorter RMST to PD diagnosis (difference, –0.09 years [95% CI, –0.12 to –0.06 years]; *P* < .001), while RLS group 1 showed a significantly longer RMST to diagnosis (difference, 0.03 years [95% CI, 0.01-0.06 years]; *P* = .003) (Table 3).

**Table 2.** Characteristics of the RLS Group 1 and RLS Group 2

Characteristic	Patients, No./total No. (%)			*P* value^b^
	Overall (N = 9919)	RLS group 1 (n = 3077)^a^	RLS group 2 (n = 6842)^a^	
Age at enrollment, mean (SD), y	50.3 (16.0)	52.6 (15.0)	49.3 (16.4)	<.001^c^
Sex				
Male	3694/9919 (37.2)	1128/3077 (36.7)	2566/6842 (37.5)	.43
Female	6225/9919 (62.8)	1949/3077 (63.3)	4276/6842 (62.5)	
Income level by insurance fee				
0-30th Percentile	2390/9919 (24.1)	744/3077 (24.2)	1646/6842 (24.1)	.67
30th-70th Percentile	3709/9919 (37.4)	1167/3077 (37.9)	2542/6842 (37.1)	
70th-100th Percentile	3820/9919 (38.5)	1166/3077 (37.9)	2654/6842 (38.8)	
Region of residence				
Rural	5521/9919 (55.7)	1756/3077 (57.1)	3765/6842 (55.0)	.06
Urban	4398/9919 (44.3)	1321/3077 (42.9)	3077/6842 (45.0)	
CCI score, mean (SD)	1.30 (1.35)	1.45 (1.45)	1.24 (1.30)	<.001^c^
BMI^d^				
<18.5	293/8865 (3.3)	105/2796 (3.8)	188/6069 (3.1)	.37
18.5 to <23	3104/8865 (35.0)	963/2796 (34.4)	2141/6069 (35.3)	
23 to <25	2190/8865 (24.7)	684/2796 (24.5)	1506/6069 (24.8)	
≥25	3278/8865 (37.0)	1044/2796 (37.3)	2234/6069 (36.8)	
Smoking^d^				
No smoking	6530/8854 (73.8)	2079/2793 (74.4)	4451/6061 (73.4)	.61
Quit	725/8854 (8.2)	222/2793 (8.0)	503/6061 (8.3)	
Smoking	1599/8854 (18.0)	492/2793 (17.6)	1107/6061 (18.3)	
Alcohol^d^				
No drinking	5959/8853 (67.3)	1920/2792 (68.8)	4039/6061 (66.6)	.05
Drinking (≥1 times/wk)	2894/8853 (32.7)	872/2792 (31.2)	2022/6061 (33.4)	
Comorbidities				
Sleep disorders^e^	598/9919 (6.0)	222/3077 (7.2)	376/6842 (5.5)	.001^c^
Iron deficiency anemia	409/9919 (4.1)	137/3077 (4.5)	272/6842 (4.0)	.29

Abbreviations: BMI, body mass index (calculated as weight in kilograms divided by height in meters squared); CCI, Charlson Comorbidity Index; RLS, restless leg syndrome.

^a^ RLS group 1 (received a dopamine agonist ≥2 times), RLS group 2 (did not receive a dopamine agonist or received a dopamine agonist only 1 time).

^b^ Continuous variables by Mann-Whitney *U* test and categorical variables by the χ^2^ test.

^c^ Statistically significant.

^d^ BMI, smoking, and alcohol had missing data; therefore, the total numbers differed.

^e^ Insomnia, hypersomnia, sleep-related breathing disorder, narcolepsy, sleepwalking, sleep tremor, and nightmare.

**Table 3.** Comparison of RMST at 15 Years Between Groups

Group	Diagnosis of PD cases, No./total No. (%)	RMST, y	RMST difference (95% CI)	*P* value
Control group	99/9919 (1.0)	14.934	[Reference]	NA
RLS group 1 (DA treated)	15/3077 (0.5)	14.968	0.034 (0.012 to 0.057)	.003
RLS group 2 (DA nontreated)	143/6842 (2.1)	14.844	−0.090 (−0.120 to −0.060)	<.001

Abbreviations: DA, dopamine agonist; NA, not applicable; PD, Parkinson disease; RMST, restricted mean survival time.

## Discussion

This study analyzed a nationwide retrospective large-sample cohort and found that RLS was associated with an increased risk of developing PD and that patients with RLS tended to receive a diagnosis of PD earlier than the control group. Subsequently, we divided the patients with RLS into 2 groups based on DA treatment and compared the time to PD diagnosis between the subgroups. The patients with RLS who were not treated with DAs exhibited a statistically significantly shorter time to PD diagnosis and a higher incidence of PD, whereas those who were treated with DAs showed a statistically significantly longer time to PD diagnosis and a lower incidence of PD. Although the RMST differences between the groups were less than 1 year, these findings may be clinically meaningful, considering the incidence of PD diagnosis.

Previous retrospective cross-sectional studies on PD and RLS focused on the presence or absence of RLS symptoms among patients with PD.^1,7,9,18,19^ These studies have reported that a significant number of patients with PD exhibit RLS symptoms and that patients with PD and RLS experience reduced sleep quality and overall quality of life. In addition, these studies suggest a correlation between RLS symptom severity and PD severity.^1,9,18,20,21,22^

In contrast, some studies have examined the development of PD after the onset of RLS.^6,23^ Szatmari et al^6^ conducted a study on a nationally representative prospective cohort of nearly 3.5 million US veterans (mean [SD] age, 60 [14] years; 93% male; median follow-up time, 7.8 years [IQR, 6.4-8.4 years]). They followed up with 100 882 PD-free patients and found 68 PD cases (0.1%; incidence rate, 1.9 per 10 000 patient-years) in the RLS-negative group and 185 incident PD cases (0.4%; incidence rate, 4.7 per 10 000 patient-years) in the RLS-positive group within the propensity score–matched cohort. The authors thus concluded that patients with RLS had more than a 2-fold higher risk of developing incident PD (hazard ratio, 2.57 [95% CI, 1.95-3.39]) compared with patients without RLS.

Wong et al^23^ conducted a study on 22 999 US male health professionals aged 40 to 75 years who were enrolled in the Health Professionals Follow-up Study over an 8-year period. The study found that men with RLS symptoms occurring more than 15 times per month had a higher risk of developing PD (adjusted relative risk, 1.47 [95% CI, 0.59-3.65]).

Women are more susceptible to conditions such as IDA and hormonal changes (eg, menopause or pregnancy),^1,2,4,5,24^ which may explain why previous epidemiologic studies have consistently reported a higher prevalence of RLS among women compared with men. Our study also observed that the RLS group consisted of mostly women. However, the 2 referenced studies exclusively enrolled men and did not conduct subgroup analyses according to DA use, which is a critical distinguishing factor of the present study.

The results of this study and the previous 2 longitudinal studies suggest that patients with RLS may have an increased likelihood of developing PD. However, while RLS may be a potential risk factor for PD, it could also represent an early manifestation of PD. Therefore, to explore this possibility, we stratified patients with RLS based on DA use and examined the incidence of PD in each group. The DA-treated group tended to be at decreased risk of developing PD, whereas the DA-nontreated group tended to be at increased risk of developing PD, suggesting that the association between RLS and PD might not be due to the dopaminergic pathway, but a different pathophysiological mechanism. Based on these findings, it may be more reasonable to interpret RLS as a potential risk factor for PD rather than an early manifestation.

Numerous studies have suggested that RLS and PD may be associated through various mechanisms other than the dopaminergic pathway.^1,3^ First, among nondopaminergic systems, the noradrenergic system could be a potential connection between RLS and PD. Several studies support the involvement of the locus coeruleus and its projections to the central nervous system in the pathologic findings of both PD and RLS.^4^ In addition, patients with RLS often experience a decline in sleep quality, and sleep disturbances could serve as a risk factor for PD.^25^ It is hypothesized that chronic intermittent hypoxia caused by sleep apnea may increase oxidative stress and inflammation, which may contribute to the pathophysiology of PD.^26,27^ In addition, the glymphatic system, which clears waste products from the brain during sleep, is activated during sleep.^28^ When sleep is insufficient, this clearance function may be impaired, leading to the accumulation of waste products such as α-synuclein in patients with PD.^29^ Finally, IDA may contribute to the association between the 2 disorders. Iron plays a role in biosynthesis and transmission of dopamine.^30^ A previous study reported that 20.8% of patients with PD presented with RLS, and lower serum ferritin levels were associated with RLS symptoms among patients with PD.^18^

There is still no consensus on whether L-dopa and other drugs such as DAs and monoamine oxidase inhibitors can slow the progression of PD.^31,32,33,34^ However, our study suggests 2 possible explanations for the finding that the incidence of PD in RLS group 2 (DA nontreated) was significantly higher than in RLS group 1 (DA treated).

First, because this study did not regard RLS to be a prodromal manifestation of PD, the use of DAs is considered to reflect a symptomatic rather than a neuroprotective effect. In contrast to this study, if RLS had been regarded as an early manifestation of PD, DAs might have been considered to exert a neuroprotective effect in preventing progression to PD.^35,36,37,38^ Second, in the DA-nontreated group, 2 possible scenarios may account for the increased incidence of PD: (1) patients may have voluntarily discontinued DAs despite their potential symptomatic effect, thereby losing their benefit, or (2) patients may have discontinued DA due to lack of effectiveness. It is possible that the DA-nontreated group included patients with conditions other than primary RLS, while most patients with primary RLS typically respond well to DA therapy.

### Strengths and Limitations

This study has some strengths. A major strength of this study is that, unlike previous studies that included predominantly male participants, it was conducted among a general population cohort.^6,23^ Furthermore, rather than simply examining the association between RLS and PD, we conducted a secondary analysis stratified by DA treatment, providing a more in-depth exploration of the association between the 2 diseases. The 2 subgroups that we operationally defined showed significant differences, thereby supporting the meaningfulness of the subgroup analysis.

This study also has several limitations. First, patients with RLS and those with PD were identified using *ICD-10* codes recorded by physicians based on clinical judgment. Therefore, there is a possibility of underdiagnosis or overdiagnosis. For example, rapid eye movement sleep behavior disorder, a well-known prodromal feature of PD, may have been misdiagnosed as RLS in some cases. Second, the subgroups were divided based on an operational definition into those who took DAs during 2 or more distinct clinical visits and those who did not. It might be difficult to completely distinguish between idiopathic RLS and secondary RLS based on DA treatment, as some patients might have discontinued treatment due to adverse effects of DAs. Third, although this study excluded individuals who received a diagnosis of PD prior to an RLS diagnosis and used a large, nationwide cohort, it was not able to confirm a causal relationship or shared dopaminergic pathway dysfunction between RLS and PD. To clarify these associations, future studies should use the National Health Insurance Service Customized Database (2002-2020), which covers more than 97% of the Korean population and allows for more comprehensive and stratified analyses.

## Conclusions

This cohort study suggests that RLS may be associated with an increased risk of developing PD. Patients with RLS who were not treated with DAs tended to have an elevated risk of developing PD, whereas those who were treated with DAs tended to have a decreased risk of developing PD. Based on these findings, it is possible that the pathophysiological bridge between RLS and PD may involve alternative mechanisms other than the dopaminergic pathway.

## Notes

Supplement 1. **eMethods.** Detailed description of statistical analysis **eTable 1.** Imbalance Statistics for Table 1 **eTable 2.** Imbalance Statistics for Table 2 **eFigure.** Plot of Imbalance Statistics Before and After Matching

Supplement 2.
Data Sharing Statement

## References

[1] Maggi G, Barone A, Mastromarino C, Santangelo G, Vitale C. Prevalence and clinical profile of patients with restless legs syndrome in Parkinson’s disease: a meta-analysis. Sleep Med. 2024;121:275-286. doi:10.1016/j.sleep.2024.07.015 39033665

[2] Berger K, Luedemann J, Trenkwalder C, John U, Kessler C. Sex and the risk of restless legs syndrome in the general population. Arch Intern Med. 2004;164(2):196-202. doi:10.1001/archinte.164.2.196 14744844

[3] Bugnicourt JM. Dopamine agonists in the treatment of restless legs syndrome: too much of a good thing? J Sleep Med. 2024;21(1):1-5. doi:10.13078/jsm.230030

[4] Verbaan D, van Rooden SM, van Hilten JJ, Rijsman RM. Prevalence and clinical profile of restless legs syndrome in Parkinson’s disease. Mov Disord. 2010;25(13):2142-2147. doi:10.1002/mds.23241 20737549

[5] Gossard TR, Trotti LM, Videnovic A, St Louis EK. Restless legs syndrome: contemporary diagnosis and treatment. Neurotherapeutics. 2021;18(1):140-155. doi:10.1007/s13311-021-01019-4 33880737 PMC8116476

[6] Szatmari S Jr, Bereczki D, Fornadi K, Kalantar-Zadeh K, Kovesdy CP, Molnar MZ. Association of restless legs syndrome with incident Parkinson’s disease. Sleep. 2017;40(2):zsw065. doi:10.1093/sleep/zsw065 28364505 PMC6084760

[7] Schrag A, Bohlken J, Dammertz L, . Widening the spectrum of risk factors, comorbidities, and prodromal features of Parkinson disease. JAMA Neurol. 2023;80(2):161-171. doi:10.1001/jamaneurol.2022.3902 36342675 PMC9641600

[8] Ferini-Strambi L, Carli G, Casoni F, Galbiati A. Restless legs syndrome and Parkinson disease: a causal relationship between the two disorders? Front Neurol. 2018;9:551. doi:10.3389/fneur.2018.00551 30087647 PMC6066514

[9] Krishnan PR, Bhatia M, Behari M. Restless legs syndrome in Parkinson’s disease: a case-controlled study. Mov Disord. 2003;18(2):181-185. doi:10.1002/mds.10307 12539212

[10] Garcia-Borreguero D, Odin P, Serrano C. Restless legs syndrome and PD: a review of the evidence for a possible association. Neurology. 2003;61(6)(suppl 3):S49-S55. doi:10.1212/WNL.61.6_suppl_3.S49 14504380

[11] Kim MK, Han K, Lee SH. Current trends of big data research using the Korean National Health Information Database. Diabetes Metab J. 2022;46(4):552-563. doi:10.4093/dmj.2022.0193 35929173 PMC9353560

[12] World Medical Association. World Medical Association Declaration of Helsinki: ethical principles for medical research involving human subjects. JAMA. 2013;310(20):2191-2194. doi:10.1001/jama.2013.281053 24141714

[13] Bae S, Hong I, Baek MS. Association between the length of stay in rehabilitation and mortality among the adults with Parkinson’s disease: 2009-2019 Korean National Health Insurance Service Databases. Front Aging Neurosci. 2024;16:1428972. doi:10.3389/fnagi.2024.1428972 39161340 PMC11330883

[14] Ho D, Imai K, King G, Stuart EA. MatchIt: nonparametric preprocessing for parametric causal inference. J Stat Softw. 2011;42:1-28. doi:10.18637/jss.v042.i08

[15] Hedges LV, Olkin I. Statistical Methods for Meta-Analysis. Academic Press; 2014.

[16] Cohen J. *Statistical Power Analysis for the Behavioral Sciences*. Routledge; 2013.

[17] Yang D, Dalton JE. A Unified Approach to Measuring the Effect Size Between Two Groups Using SAS. Citeseer; 2012:1-6.

[18] Ondo WG, Vuong KD, Jankovic J. Exploring the relationship between Parkinson disease and restless legs syndrome. Arch Neurol. 2002;59(3):421-424. doi:10.1001/archneur.59.3.421 11890847

[19] Piao YS, Lian TH, Hu Y, . Restless legs syndrome in Parkinson disease: clinical characteristics, abnormal iron metabolism and altered neurotransmitters. Sci Rep. 2017;7(1):10547. doi:10.1038/s41598-017-10593-7 28874701 PMC5585207

[20] Minar M, Kosutzka Z, Danterova K, et al. Restless legs syndrome in Parkinson’s disease: relationship with quality of life and medication. Bratisl Lek Listy. 2022;123(1):55-60. doi:10.4149/BLL_2022_009 34967659

[21] Gao X, Schwarzschild MA, O’Reilly EJ, Wang H, Ascherio A. Restless legs syndrome and Parkinson’s disease in men. Mov Disord. 2010;25(15):2654-2657. doi:10.1002/mds.23256 20737545 PMC3114885

[22] Peeraully T, Tan EK. Linking restless legs syndrome with Parkinson’s disease: clinical, imaging and genetic evidence. Transl Neurodegener. 2012;1(1):6. doi:10.1186/2047-9158-1-6 23211049 PMC3514082

[23] Wong JC, Li Y, Schwarzschild MA, Ascherio A, Gao X. Restless legs syndrome: an early clinical feature of Parkinson disease in men. Sleep. 2014;37(2):369-372. doi:10.5665/sleep.3416 24497665 PMC3900617

[24] Wesström J, Nilsson S, Sundström-Poromaa I, Ulfberg J. Restless legs syndrome among women: prevalence, co-morbidity and possible relationship to menopause. Climacteric. 2008;11(5):422-428. doi:10.1080/13697130802359683 18781488

[25] Bohnen NI, Hu MTM. Sleep disturbance as potential risk and progression factor for Parkinson’s disease. J Parkinsons Dis. 2019;9(3):603-614. doi:10.3233/JPD-191627 31227656 PMC6700634

[26] Lurie A. Inflammation, oxidative stress, and procoagulant and thrombotic activity in adults with obstructive sleep apnea. In: Obstructive Sleep Apnea in Adults. Karger Publishers; 2011:43-66. doi:10.1159/000325105 22005189

[27] Kaminska M, Lafontaine AL, Kimoff RJ. The interaction between obstructive sleep apnea and Parkinson’s disease: possible mechanisms and implications for cognitive function. Parkinsons Dis. 2015;2015(1):849472. doi:10.1155/2015/849472 26509097 PMC4609874

[28] Iliff JJ, Wang M, Liao Y, . A paravascular pathway facilitates CSF flow through the brain parenchyma and the clearance of interstitial solutes, including amyloid β. Sci Transl Med. 2012;4(147):147ra111. doi:10.1126/scitranslmed.3003748 22896675 PMC3551275

[29] Xie L, Kang H, Xu Q, . Sleep drives metabolite clearance from the adult brain. Science. 2013;342(6156):373-377. doi:10.1126/science.1241224 24136970 PMC3880190

[30] Youdim MB, Ben-Shachar D, Ashkenazi R, Yehuda S. Brain iron and dopamine receptor function. Adv Biochem Psychopharmacol. 1983;37:309-321. 6138953

[31] Group PS; Parkinson Study Group. A controlled trial of rasagiline in early Parkinson disease: the TEMPO Study. Arch Neurol. 2002;59(12):1937-1943. doi:10.1001/archneur.59.12.1937 12470183

[32] Rascol O, Brooks DJ, Korczyn AD, De Deyn PP, Clarke CE, Lang AE. A five-year study of the incidence of dyskinesia in patients with early Parkinson’s disease who were treated with ropinirole or levodopa. N Engl J Med. 2000;342(20):1484-1491. doi:10.1056/NEJM200005183422004 10816186

[33] Ahlskog JE, Muenter MD. Frequency of levodopa-related dyskinesias and motor fluctuations as estimated from the cumulative literature. Mov Disord. 2001;16(3):448-458. doi:10.1002/mds.1090 11391738

[34] Olanow CW, Kieburtz K, Schapira AH. Why have we failed to achieve neuroprotection in Parkinson’s disease? Ann Neurol. 2008;64(S2)(suppl 2):S101-S110. doi:10.1002/ana.21461 19127580

[35] Le WD, Jankovic J. Are dopamine receptor agonists neuroprotective in Parkinson’s disease? Drugs Aging. 2001;18(6):389-396. doi:10.2165/00002512-200118060-00001 11419913

[36] Schapira AH. Neuroprotection and dopamine agonists. Neurology. 2002;58(4)(suppl 1):S9-S18. 11909981 10.1212/wnl.58.suppl_1.s9

[37] Schapira AH. Dopamine agonists and neuroprotection in Parkinson’s disease. Eur J Neurol. 2002;9(suppl 3):7-14. doi:10.1046/j.1468-1331.9.s3.9.x 12464116

[38] Pringsheim T, Day GS, Smith DB, ; Guideline Subcommittee of the AAN. Dopaminergic therapy for motor symptoms in early Parkinson disease practice guideline summary: a report of the AAN guideline subcommittee. Neurology. 2021;97(20):942-957. doi:10.1212/WNL.0000000000012868 34782410 PMC8672433

