# Supplementary material for: Risk of Parkinson Disease Among Patients With Restless Leg Syndrome
Source: JAMA Netw Open. 2025 Oct 6;8(10):e2535759. doi: 10.1001/jamanetworkopen.2025.35759 (PMC12501809; doi:10.1001/jamanetworkopen.2025.35759)
Supplement: Supplement 1. — eMethods. Detailed description of statistical analysis eTable 1. Imbalance Statistics for Table 1 eTable 2. Imbalance Statistics for Table 2 eFigure. Plot of Imbalance Statistics Before and After Matching [file jamanetwopen-e2535759-s001.pdf]

Supplementary Online Content

Bang M, Park D, Kim JH, Kim HS. Risk of Parkinson disease among patients with restless leg syndrome. *JAMA Netw Open.* 2025;8(10):e2535759. doi:10.1001/jamanetworkopen.2025.35759

eMethods. Detailed Description of Statistical Analysis
eTable 1. Imbalance Statistics for Table 1
eTable 2. Imbalance Statistics for Table 2
eFigure. Plot of Imbalance Statistics Before and After Matching

This supplementary material has been provided by the authors to give readers additional information about their work.

© 2025 Bang M et al. *JAMA Network Open.*

eMethods. Detailed Description of Statistical Analysis

Baseline characteristics were compared using χ^2 tests for categorical variables and Mann-Whitney U tests for continuous variables.

Standardized Mean Differences (SMDs), variance ratios and empirical cumulative distribution function(eCDF) statistics were estimated using the R package MatchIt.[13] The SMDs and their 95% confidence intervals (CIs) were computed as follows: for continuous variables, CIs were calculated based on the small-sample bias-corrected effect size (Hedges' g) and its variance formula as proposed by Hedges and Olkin[14]; for binary variables, CIs were derived using the standardized mean difference for proportions with variance estimation following Cohen's approach for effect sizes between proportions[15]; and for multi-level categorical variables, the Mahalanobis distance-based extension was used.[16] A Unified Approach to Measuring the Effect Size Between Two Groups Using SAS®. SAS Global Forum.).

All participants who are free of Parkinson's disease (PD) and RLS were followed from 1 January 2004 until the first occurrence of PD, death, or 31 December 2019(whichever came first). RLS was treated as a time-varying approach: for individuals who eventually developed RLS, person-time from baseline up to the date of the first RLS diagnosis was classified as control, and person-time thereafter was classified as exposed. By assigning the pre-diagnosis interval to the control group, this data structure eliminates immortal-time bias. To control for confounding by baseline characteristics, we implemented an inverse probability of treatment weighting (IPTW) method. Propensity scores, representing the predicted probability of receiving an RLS diagnosis given a set of baseline covariates, were estimated for each subject using a logistic regression model with covariates (age, sex, income, region, CCI score, BMI, smoking, alcohol, history of sleep disorder, iron deficiency anemia) to obtain each propensity scores(PS), i.e. the predicted probability of being diagnosed with RLS at or before each person-

© 2025 Bang M et al. *JAMA Network Open.*

time segment. To enhance statistical stability and avoid the influence of extreme weights, we calculated stabilized weights (SW). For every person-time interval i, a stabilised weight was calculated as

$$\omega_i = \frac{P_r(T = t_i)}{P_r(T = t_i | X_i)}$$

where T denotes the observed exposure status in the interval ($t_i = 1$ for RLS, $t_i = 0$ for control) and X_i the covariate vector. Hence

$$\omega_i = \begin{cases} \dfrac{Pr(T = 1)}{PS_i}, & if\ interval\ is\ exposed \\ \dfrac{Pr(T = 0)}{1 - PS_i}, & if\ interval\ is\ unexposed \end{cases}$$

This formulation preserves the marginal exposure proportion in the numerator while down-weighting observations that were either very unlikely or very likely to receive the exposure given their covariates. To further mitigate the impact of outlier weights, we truncated the stabilized weights distribution at the 5th and 95th percentiles: values below the 5th percentile were set equal to the 5th-percentile value, and values above the 95th percentile were set equal to the 95th-percentile value. All subsequent time-to-event analyses were carried out with these trimmed stabilised weights. Since the proportional hazards assumption was violated (Schoenfeld residual test, $p < 0.001$), we estimated the restricted mean survival time (RMST) as a robust alternative to the hazard ratio.

© 2025 Bang M et al. *JAMA Network Open.*

eTable 1. Imbalance Statistics for Table 1

	SMD	SMD 95% CI lower	SMD 95% CI upper	Variance Ratio	eCDF mean	eCDF max
**Age at enrollment (years)**	0.012	-0.016	0.04	0.9628	0.004	0.019
**Sex**						
Male	0	NA	NA	NA	0	0
Female	0	NA	NA	NA	0	0
**Income level by insurance fee**						
Bottom 0 to 30th percentile	0	NA	NA	NA	0	0
Bottom 30th to 70th percentile	0	NA	NA	NA	0	0
Bottom 70th to 100th percentile	0	NA	NA	NA	0	0
**Region of residence**						
Rural	0	NA	NA	NA	0	0
Urban	0	NA	NA	NA	0	0
**CCI score**	0	NA	NA	1	0	0
**BMI**						
<18.5	-0.009	-0.039	0.021	NA	0.002	0.002
<23	-0.047	-0.077	-0.017	NA	0.022	0.022
<25	0.013	-0.016	0.043	NA	0.006	0.006
>=25	0.038	0.008	0.068	NA	0.018	0.018
**Smoking**						
No smoking	-0.012	-0.041	0.018	NA	0.005	0.005
Quit	-0.011	-0.041	0.019	NA	0.003	0.003
Smoking	0.021	-0.009	0.051	NA	0.008	0.008
**Alcohol**						
No drinking	0.076	0.046	0.106	NA	0.036	0.036
Drinking(1 or more per week	-0.076	-0.106	-0.046	NA	0.036	0.036
**Comorbidities**						

© 2025 Bang M et al. *JAMA Network Open.*

**Sleep disorders**						
None	-0.089	-0.117	-0.061	NA	0.021	0.021
Yes	0.089	0.061	0.117	NA	0.021	0.021
**Iron deficiency anemia**						
None	-0.027	-0.055	0.001	NA	0.005	0.005
Yes	0.027	-0.001	0.055	NA	0.005	0.005

SMD, standardized mean differences; eCDF, empirical cumulative distribution function; NA, not applicable

© 2025 Bang M et al. *JAMA Network Open.*

eTable 2. Imbalance Statistics for Table 2

	SMD	SMD 95% CI lower	SMD 95% CI upper	Variance Ratio	eCDF mean	eCDF max
**Age at enrollment (years)**	0.244	0.214	0.275	0.821	0.038	0.0861
**Sex**						
Male	-0.018	-0.049	0.012	NA	0.009	0.009
Female	0.018	-0.012	0.049	NA	0.009	0.009
**Income level by insurance fee**						
Bottom 0 to 30th percentile	0.0023	-0.028	0.033	NA	0.001	0.001
Bottom 30th to 70th percentile	0.021	-0.001	0.051	NA	0.01	0.01
Bottom 70th to 100th percentile	-0.023	-0.053	0.008	NA	0.011	0.011
**Region of residence**						
Rural	0.035	0.005	0.066	NA	0.018	0.018
Urban	-0.035	-0.066	-0.005	NA	0.018	0.018
**CCI score**	0.153	0.123	0.183	1.261	0.017	0.066
**BMI**						
<18.5	0.034	0.002	0.066	NA	0.007	0.007
<23	-0.023	-0.055	0.009	NA	0.011	0.011
<25	-0.002	-0.034	0.03	NA	0.001	0.001
>=25	0.011	-0.021	0.042	NA	0.005	0.005
**Smoking**						
No smoking	0.023	-0.009	0.055	NA	0.01	0.01
Quit	-0.007	-0.038	0.025	NA	0.002	0.002
Smoking	-0.022	-0.054	0.001	NA	0.008	0.008
**Alcohol**						
No drinking	0.053	0.021	0.085	NA	0.025	0.025
Drinking(1 or more per week	-0.053	-0.085	-0.021	NA	0.025	0.025
**Comorbidities**						

© 2025 Bang M et al. *JAMA Network Open.*

**Sleep disorders**						
None	-0.07	-0.101	-0.039	NA	0.018	0.018
Yes	0.07	0.039	0.101	NA	0.018	0.018
**Iron deficiency anemia**						
None	-0.027	-0.057	0.004	NA	0.006	0.006
Yes	0.027	-0.004	0.057	NA	0.006	0.006

SMD, standardized mean differences; eCDF, empirical cumulative distribution function; NA, not applicable

© 2025 Bang M et al. *JAMA Network Open.*

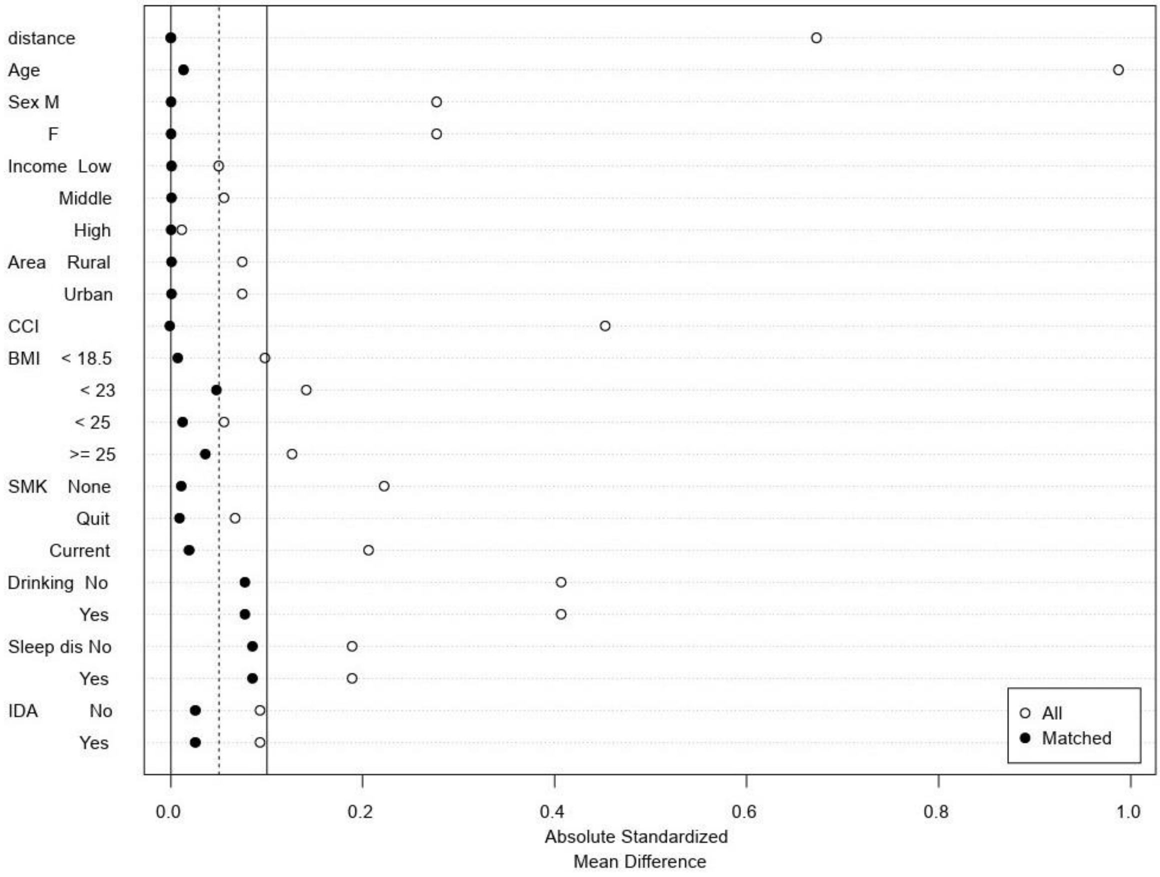

eFigure. Plot of Imbalance Statistics Before and After Matching

© 2025 Bang M et al. *JAMA Network Open.*
