# Supplementary material for: Risk of Parkinson Disease Among Patients With Restless Leg Syndrome
Source: JAMA Netw Open. 2025 Oct 6;8(10):e2535759. doi: 10.1001/jamanetworkopen.2025.35759 (PMC12501809; doi:10.1001/jamanetworkopen.2025.35759)
Supplement: Supplement 2. — Data Sharing Statement [file jamanetwopen-e2535759-s002.pdf]

Data Sharing Statement

Bang. Risk of Parkinson Disease Among Patients With Restless Leg Syndrome. *JAMA Netw Open*. Published October 06, 2025. doi:10.1001/jamanetworkopen.2025.35759

Data
Data available: No

Additional Information
Explanation for why data not available: According to the Korean public health data policy, Access to data cannot be open to unauthorized persons.
